# Supplementary material for: Transcriptomic profiling of skeletal muscle adaptations to exercise and inactivity
Source: Nat Commun. 2020 Jan 24;11:470. doi: 10.1038/s41467-019-13869-w (PMC6981202; doi:10.1038/s41467-019-13869-w)
Supplement: Supplementary file 1 — Supplementary Information [file 41467_2019_13869_MOESM1_ESM.pdf]

Transcriptomic Profiling of Skeletal Muscle Adaptations to Exercise and Inactivity

Pillon et al.

Supplementary information

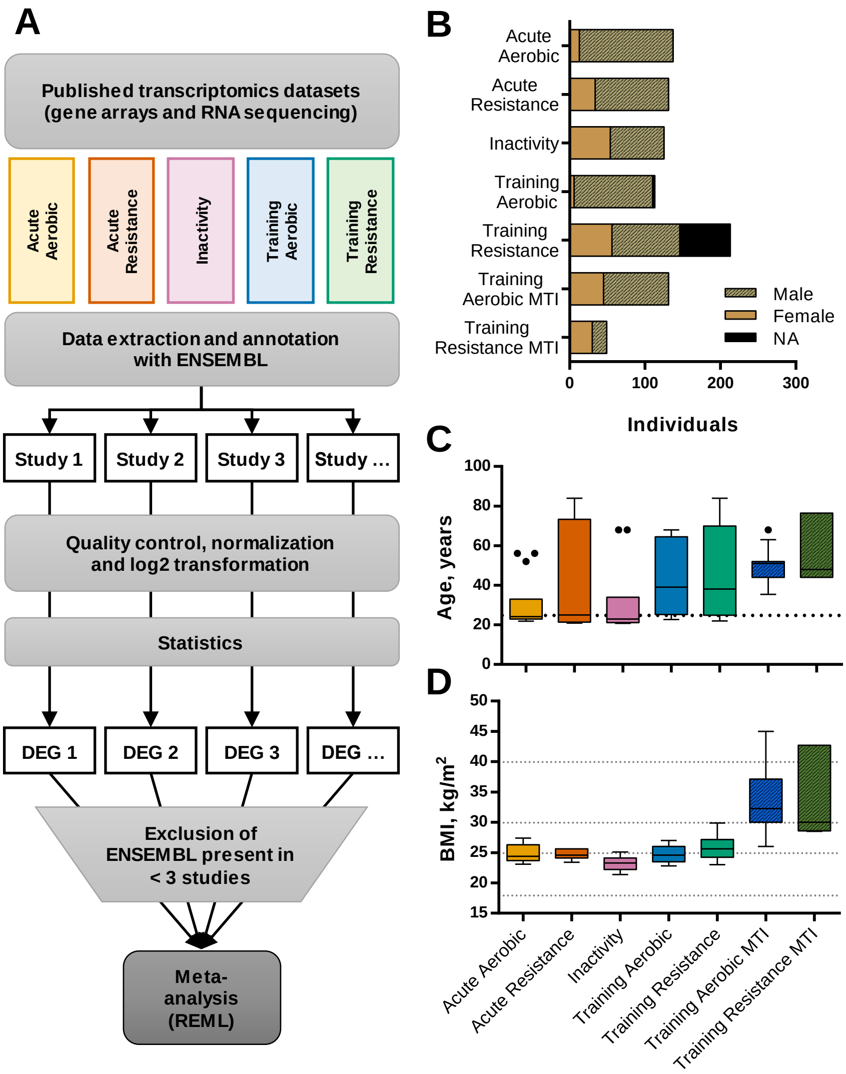

Supplementary Figure 1. Workflow and clinical characteristics. The study design is presented for all 5 categories of acute, training and inactivity studies (A). The same methodology was applied to all studies: collection of raw data when available, quality control, annotation, normalization and calculation of statistics (fold-change, FDR). The meta-analysis was then calculated using the restricted maximum-likelihood method taking into account the average, standard deviation and sample size for each study. Studies had a minority of female participants (B) and various age (C). Body mass index (D) was similar for studies of healthy individuals, but significantly higher in studies that included metabolically impaired (MTI) individuals. Tukey box and whiskers plots. DEG: Differentially Expressed Genes.

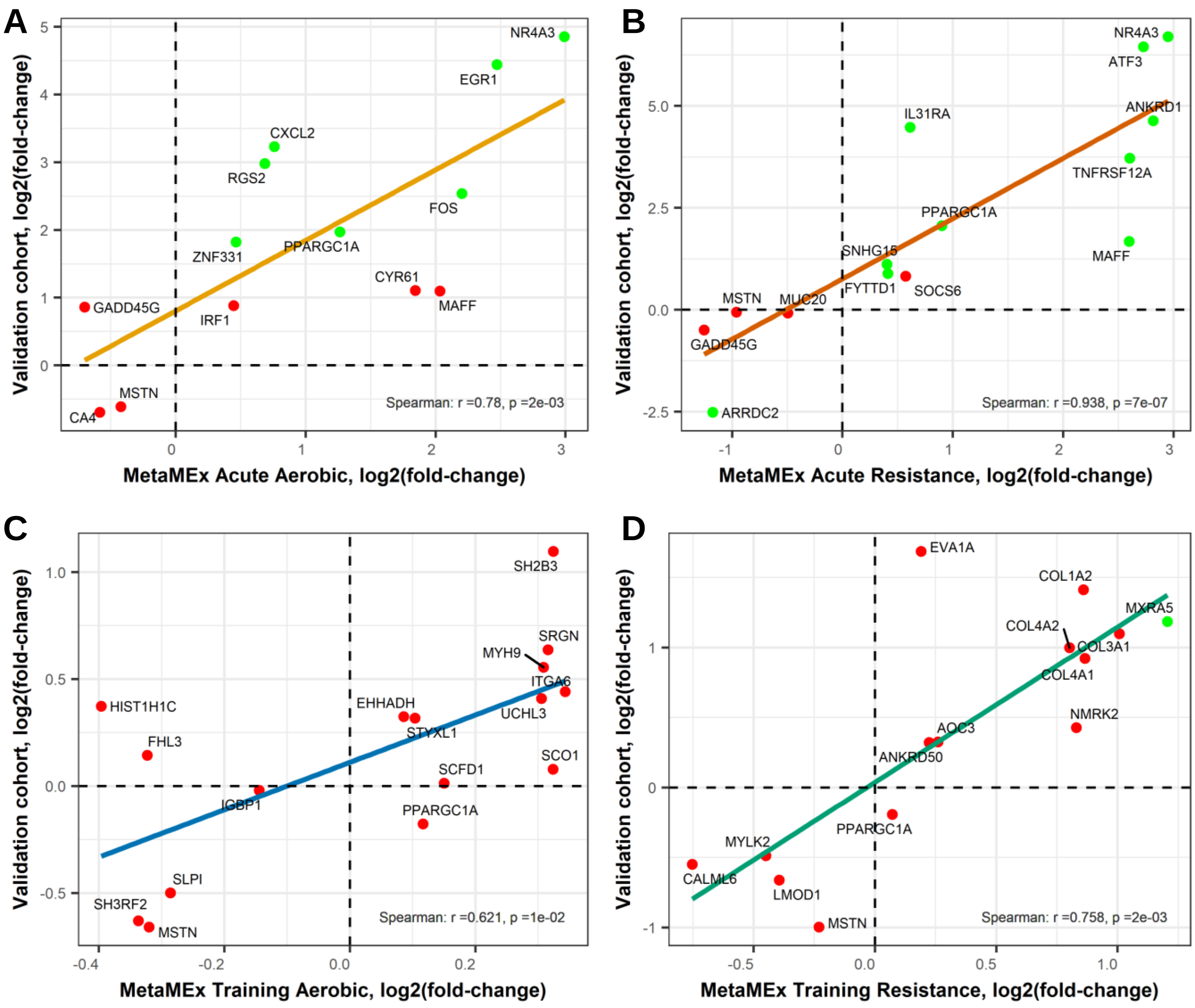

Supplementary Figure 2. Validation of the meta-analysis using independent exercise cohorts. Correlation of the top genes found significantly changed in exercise in MetaMEx with mRNA expression, as assessed by qPCR, in biopsy samples obtained from independent cohorts of acute aerobic (A), acute resistance (B), aerobic training (C) and resistance training (D). Red dots represent genes that do not reach significance in the validation cohorts (n=8, p<0.05).

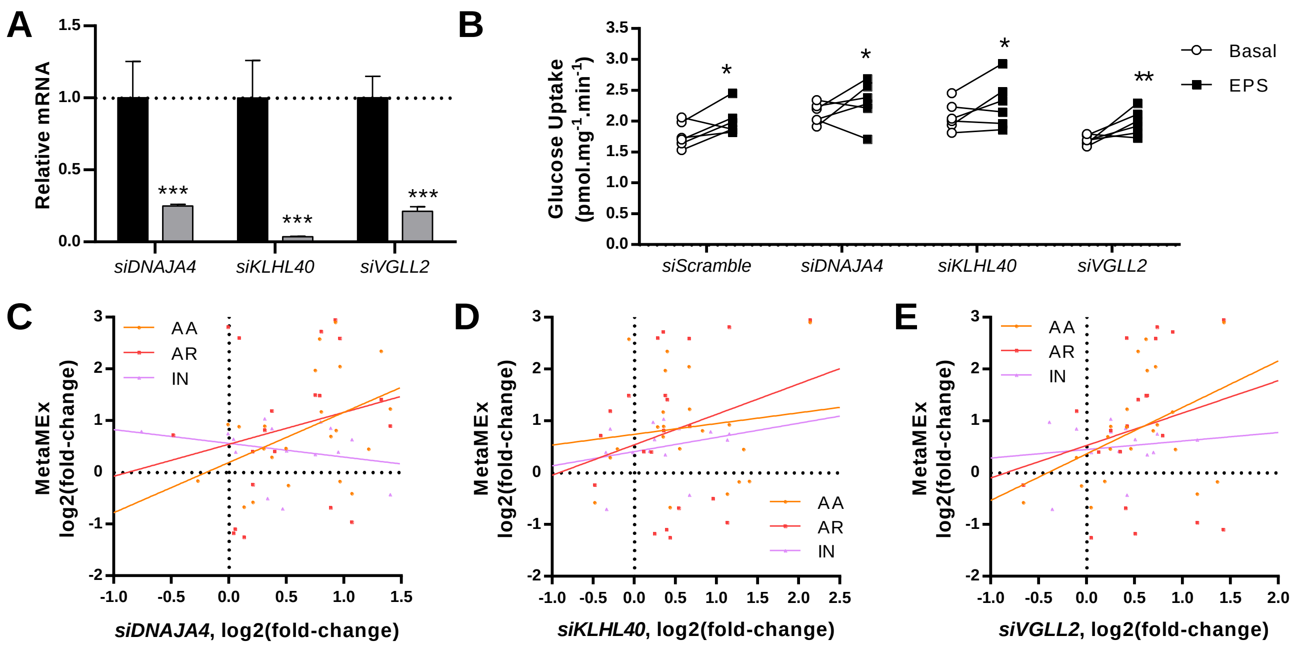

Supplementary Figure 3. Selection pipeline of exercise and inactivity responsive genes. A) Silencing efficiency using siRNA against *DNAJA4*, *KLHL40* and *VGLL2*. Data are mean ±SEM, individual paired t-test vs scramble, ***p<0.001 B) Electrical pulse stimulation-induced glucose uptake after silencing of *DNAJA4*, *KLHL40* and *VGLL2*. 2-way ANOVA (siRNA, EPS), *n*=6, *p<0.05, **p<0.01. C-E) Exercise and inactivity responsive genes selected in MetaMEx were correlated with their expression level in primary human muscle cells after silencing of *DNAJA4*, *KLHL40* or *VGLL2*.

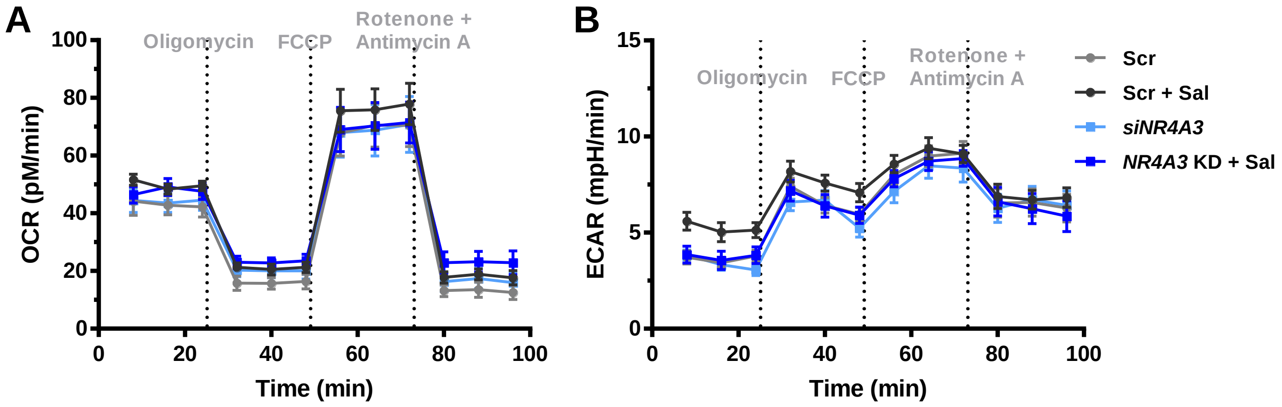

Supplementary Figure 4. Seahorse XF analysis of primary skeletal muscle cells exposed to the beta-adrenergic stimulator salbutamol and *NR4A3* silencing. *NR4A3* was silenced using siRNA. Cells were exposed to 20 µM of salbutamol for 3h before analyzing oxygen consumption and extracellular acidification rate using the seahorse technology.

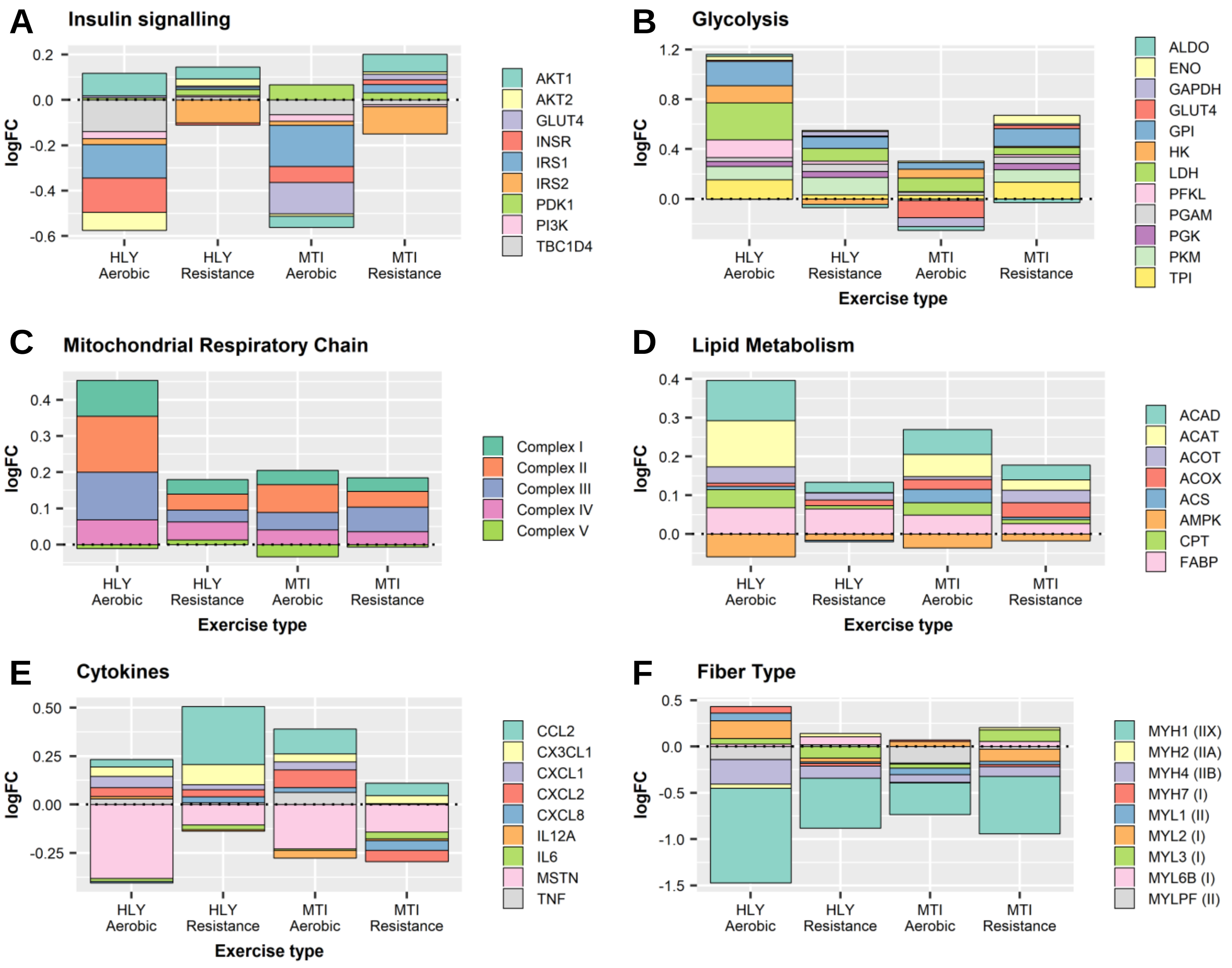

Supplementary Figure 5. Pathways differentially regulated by exercise in metabolically impaired individuals. Genes corresponding to the proteins of interest were collected from the KEGG database and fold-changes were added to present the overall modification of enzymes involved in pathways. The behavior of insulin signaling (A), glycolysis (B), mitochondrial respiration (C), lipid metabolism signaling (D), inflammation (E) and muscle fiber composition (F) are presented.

Supplementary Table 1. TaqMan primers.

Gene Symbol(s)	Assay ID	Gene Name(s)	Gene Alias(es)
**18S**	Hs99999901_s1	Eukaryotic 18S rRNA	-
**ADAM12**	Hs01106101_m1	ADAM metallopeptidase domain 12	ADAM12-OT1;CAR10;MCMP;MCMPMltna; MLTN;MLTNA
**ADH1C**	Hs02383872_s1	alcohol dehydrogenase 1C (class I), gamma polypeptide	ADH3
**AMOTL2**	Hs01048101_m1	angiomotin like 2	LCCP
**ANKRD1**	Hs00173317_m1	ankyrin repeat domain 1	ALRP;C-193;CARP;CVARP;MCARP;bA320F15.2
**ANKRD50**	Hs00379454_m1	ankyrin repeat domain 50	-
**AOC3**	Hs02560271_s1	amine oxidase, copper containing 3	HPAO;SSAO;VAP-1;VAP1
**ARRDC2**	Hs00367526_g1	arrestin domain containing 2	CLONE24945;PP2703
**ATF3**	Hs00231069_m1	activating transcription factor 3	-
**AVIL**	Hs00198114_m1	advillin	ADVIL;DOC6;p92
**B2M**	Hs00187842_m1	beta-2-microglobulin	IMD43
**BGN**	Hs00959143_m1	biglycan	DSPG1;PG-S1;PGI;SLRR1A
**BRSK2**	Hs00908871_m1	BR serine/threonine kinase 2	C11orf7;PEN11B;SAD1;SADA;STK29
**C2**	Hs00918862_m1	complement component 2	ARMD14;CO2
**CA14**	Hs01077605_g1	carbonic anhydrase 14	CAXiV
**CA4**	Hs00426343_m1	carbonic anhydrase 4	CAIV;Car4;RP17
**CALML6**	Hs00758338_g1	calmodulin like 6	CAGLP
**CAPN6**	Hs00560073_m1	calpain 6	CANPX;CAPNX;CalpM;DJ914P14.1
**CARD10**	Hs00367225_m1	caspase recruitment domain family member 10	BIMP1;CARMA3
**CDC25B**	Hs01582335_m1	cell division cycle 25B	-
**CDK2**	Hs01548894_m1	cyclin dependent kinase 2	CDKN2;p33(CDK2)
**CHAF1B**	Hs01123302_m1	chromatin assembly factor 1 subunit B	CAF-1;CAF-IP60;CAF1;CAF1A;CAF1P60; MPHOSPH7;MPP7
**CHRDL2**	Hs01060234_m1	chordin like 2	BNF1;CHL2
**CHRNA1**	Hs00909664_m1	cholinergic receptor nicotinic alpha 1 subunit	ACHRA;ACHRD;CHRNA;CMS1A;CMS1B; CMS2A;FCCMS;SCCMS
**CHRND**	Hs00897937_m1	cholinergic receptor nicotinic delta subunit	ACHRD;CMS2A;CMS3A;CMS3B;CMS3C; FCCMS;SCCMS
**CIART**	Hs00328968_m1	circadian associated repressor of transcription	C1orf51;CHRONO;GM129
**COL1A2**	Hs01028956_m1	collagen type I alpha 2 chain	OI4
**COL25A1**	Hs00930851_m1	collagen type XXV alpha 1	AMY;CFEOM5;CLAC;CLAC-P;CLACP
**COL3A1**	Hs00943809_m1	collagen type III alpha 1 chain	EDS4A
**COL4A1**	Hs00266237_m1	collagen type IV alpha 1 chain	BSVD;RATOR
**COL4A2**	Hs05006309_m1	collagen type IV alpha 2	ICH;POREN2
**COL6A3**	Hs00915125_m1	collagen type VI alpha 3 chain	BTHLM1;DYT27;UCMD1
**CXCL2**	Hs00601975_m1	C-X-C motif chemokine ligand 2	CINC-2a;GRO2;GROb;MGSA-b;MIP-2a; MIP2;MIP2A;SCYB2
**CYP1B1**	Hs00164383_m1	cytochrome P450 family 1 subfamily B member 1	CP1B;CYPIB1;GLC3A;P4501B1
**CYP4B1**	Hs01086311_m1	cytochrome P450 family 4 subfamily B member 1	CYPIVB1;P-450HP
**CYR61**	Hs00155479_m1	cysteine rich angiogenic inducer 61	CCN1;GIG1;IGFBP10
**DDIT4**	Hs01111686_g1	DNA damage inducible transcript 4	Dig2;REDD-1;REDD1
**DNAJA4**	Hs00388055_m1	DnaJ heat shock protein family (Hsp40) member A4	MST104;MSTP104;PRO1472
**DTL**	Hs00978565_m1	denticleless E3 ubiquitin protein ligase homolog	CDT2;DCAF2;L2DTL;RAMP
**EGR1**	Hs00152928_m1	early growth response 1	AT225;G0S30;KROX-24;NGFI-A;TIS8; ZIF-268;ZNF225
**EHHADH**	Hs00157347_m1	enoyl-CoA, hydratase/3-hydroxyacyl CoA dehydrogenase	ECHD;FRTS3;L-PBE;LBFP;LBP;PBFE
**EMCN**	Hs01038204_m1	endomucin	EMCN2;MUC14
**EVA1A**	Hs00259924_m1	eva-1 homolog A, regulator of programmed cell death	FAM176A;TMEM166
**EXTL1**	Hs00184929_m1	exostosin like glycosyltransferase 1	EXTL
**FABP4**	Hs01086177_m1	fatty acid binding protein 4	A-FABP;AFABP;ALBP;HEL-S-104;aP2
**FHL3**	Hs00916408_g1	four and a half LIM domains 3	SLIM2
**FLRT3**	Hs01922255_s1	fibronectin leucine rich transmembrane protein 3	HH21
**FMO2**	Hs01025544_m1	flavin containing monooxygenase 2	FMO1B1
**FOS**	Hs04194186_s1	Fos proto-oncogene, AP-1 transcription factor subunit	AP-1;C-FOS;p55
**FSIP1**	Hs00401705_m1	fibrous sheath interacting protein 1	HSD10
**FYTTD1**	Hs00260265_m1	forty-two-three domain containing 1	UIF
**GADD45A**	Hs00169255_m1	growth arrest and DNA damage inducible alpha	DDIT1;GADD45
**GADD45G**	Hs02566147_s1	growth arrest and DNA damage inducible gamma	CR6;DDIT2;GADD45gamma;GRP17
**GAPDH**	Hs99999905_m1	glyceraldehyde-3-phosphate dehydrogenase	G3PD;GAPD;HEL-S-162eP
**GLDC**	Hs01580591_m1	glycine decarboxylase	GCE;GCSP;HYGN1
**GNRHR2**	Hs00977373_g1	gonadotropin releasing hormone receptor 2 (pseudogene)	GnRH-II-R
**HIST1H1C**	Hs00271185_s1	histone cluster 1, H1c	H1.2;H1C;H1F2;H1s-1
**HPRT1**	Hs02800695_m1	hypoxanthine phosphoribosyltransferase 1	HGPRT;HPRT
**IGBP1**	Hs00426831_mH	immunoglobulin (CD79A) binding protein 1	ALPHA-4;IBP1
**IL31RA**	Hs00371172_m1	interleukin 31 receptor A	CRL;CRL3;GLM-R;GLMR;GPL;IL-31RA; PLCA2;PRO21384;hGLM-R
**IRF1**	Hs00971965_m1	interferon regulatory factor 1	IRF-1;MAR
**ITGA6**	Hs01041011_m1	integrin subunit alpha 6	CD49f;ITGA6B;VLA-6
**KCNV2**	Hs00377936_m1	potassium voltage-gated channel modifier subfamily V member 2	KV11.1;Kv8.2;RCD3B
**KDR**	Hs00911700_m1	kinase insert domain receptor	CD309;FLK1;VEGFR;VEGFR2
**KLHL40**	Hs00328078_m1	kelch like family member 40	KBTBD5;NEM8;SRYP;SYRP
**LAMB1**	Hs01055960_m1	laminin subunit beta 1	CLM;LIS5
**LMOD1**	Hs00201704_m1	leiomodin 1	1D;64kD;D1;SM-LMOD;SMLMOD
**LRRC66**	Hs02386735_m1	leucine rich repeat containing 66	-
**LYG2**	Hs00545618_m1	lysozyme g2	LYGH;LYSG2
**MAFF**	Hs05026540_g1	MAF bZIP transcription factor F	U-MAF;hMafF
**METTL7B**	Hs00378551_m1	methyltransferase like 7B	ALDI
**MSTN**	Hs00976237_m1	myostatin	GDF8;MSLHP
**MTFP1**	Hs01062596_m1	mitochondrial fission process 1	HSPC242;MTP18
**MUC20**	Hs00416321_m1	mucin 20, cell surface associated	MUC-20
**MXRA5**	Hs01019147_m1	matrix remodeling associated 5	-
**MYH8**	Hs00267293_m1	myosin heavy chain 8	DA7;MyHC-peri;MyHC-pn;gtMHC-F
**MYH9**	Hs00159522_m1	myosin, heavy chain 9, non-muscle	BDPLT6;DFNA17;EPSTS;FTNS;MHA;

			NMHC-II-A;NMMHC-IIA;NMMHCA
**MYLK2**	Hs00263888_m1	myosin light chain kinase 2	KMLC;MLCK;MLCK2;skMLCK
**NMRK2**	Hs01043681_m1	nicotinamide riboside kinase 2	ITGB1BP3;MIBP;NRK2
**NR4A3**	Hs00545009_g1	nuclear receptor subfamily 4 group A member 3	CHN;CSMF;MINOR;NOR1;TEC
**NRAP**	Hs00328987_m1	nebulin related anchoring protein	N-RAP
**PAIP2B**	Hs04965774_m1	poly(A) binding protein interacting protein 2B	-
**PER1**	Hs00242988_m1	period circadian clock 1	PER;RIGUI;hPER
**PFKFB3**	Hs00998698_m1	6-phosphofructo-2-kinase/fructose-2,6-biphosphatase 3	IPFK2;PFK2;iPFK-2
**PPARGC1A**	Hs00173304_m1	PPARG coactivator 1 alpha	LEM6;PGC-1(alpha);PGC-1alpha;PGC-1v; PGC1;PGC1A;PPARGC1
**PPP6C**	Hs00254827_m1	protein phosphatase 6 catalytic subunit	PP6;PP6C
**PRSS50**	Hs00203179_m1	protease, serine 50	CT20;TSP50
**RASA4**	Hs04992021_m1	RAS p21 protein activator 4	CAPRI;GAPL
**RGS2**	Hs01009070_g1	regulator of G-protein signaling 2	G0S8
**RORA**	Hs00536545_m1	RAR related orphan receptor A	NR1F1;ROR1;ROR2;ROR3;RZR-ALPHA;RZRA
**RYR3**	Hs00168821_m1	ryanodine receptor 3	RYR-3
**SCFD1**	Hs00910148_m1	sec1 family domain containing 1	C14orf163;RA410;SLY1;SLY1P;STXBP1L2
**SCN1B**	Hs00962350_m1	sodium voltage-gated channel beta subunit 1	ATFB13;BRGDA5;GEFSP1
**SCO1**	Hs01552201_m1	SCO1 cytochrome c oxidase assembly protein	SCOD1
**SH2B3**	Hs01081959_g1	SH2B adaptor protein 3	IDDM20;LNK
**SH3RF2**	Hs00400148_m1	SH3 domain containing ring finger 2	HEPP1;POSHER;PPP1R39;RNF158
**SLC16A3**	Hs00358829_m1	solute carrier family 16 member 3	MCT 3;MCT 4;MCT-3;MCT-4;MCT3;MCT4
**SLC38A4**	Hs00394339_m1	solute carrier family 38 member 4	ATA3;NAT3;PAAT;SNAT4
**SLC5A1**	Hs01573793_m1	solute carrier family 5 member 1	D22S675;NAGT;SGLT1
**SLPI**	Hs00268204_m1	secretory leukocyte peptidase inhibitor	ALK1;ALP;BLPI;HUSI;HUSI-I;MPI; WAP4;WFDC4
**SMCO1**	Hs03004796_m1	single-pass membrane protein with coiled-coil domains 1	C3orf43
**SMTNL1**	Hs00418171_m1	smoothelin like 1	CHASM
**SNHG15**	Hs05031784_s1	small nucleolar RNA host gene 15	C7orf40;Linc-Myo1g;MYO1GUT
**SOCS6**	Hs04986635_s1	suppressor of cytokine signaling 6	CIS-4;CIS4;HSPC060;SOCS-4;SOCS-6; SOCS4;SSI4;STAI4;STATI4
**SRGN**	Hs01004159_m1	serglycin	PPG;PRG;PRG1
**ST7-OT4**	Hs04989394_g1	ST7 overlapping transcript 4	NCRNA00042;ST7OT4
**STYXL1**	Hs01553273_m1	serine/threonine/tyrosine interacting-like 1	DUSP24;MK-STYX;MKSTYX
**TBP**	Hs00427620_m1	TATA-box binding protein	GTF2D;GTF2D1;HDL4;SCA17;TFIID
**THAP12**	Hs01566400_g1	THAP domain containing 12	DAP4;P52rIPK;PRKRIR;THAP0
**THBS4**	Hs00170261_m1	thrombospondin 4	TSP-4;TSP4
**TMEM120A**	Hs00230518_m1	transmembrane protein 120A	NET29;TMPIT
**TMEM253**	Hs04188762_g1	transmembrane protein 253	C14orf176;C14orf95;NCRNA00220
**TNFRSF12A**	Hs00171993_m1	TNF receptor superfamily member 12A	CD266;FN14;TWEAKR
**TPRG1**	Hs00415626_m1	tumor protein p63 regulated 1	FAM79B
**UCHL3**	Hs04334565_m1	ubiquitin C-terminal hydrolase L3	UCH-L3
**UCK2**	Hs00989900_m1	uridine-cytidine kinase 2	TSA903;UK;UMPK
**VAMP5**	Hs05042428_s1	vesicle associated membrane protein 5	-
**VGLL2**	Hs00403461_m1	vestigial like family member 2	VGL2;VITO1
**ZNF331**	Hs00367929_m1	zinc finger protein 331	RITA;ZNF361;ZNF463
**ZNF784**	Hs01385515_mH	zinc finger protein 784	-

Supplementary Table 2. Description of the studies included in the analysis of healthy (HLY) and metabolically impaired (MTI) individuals.

	Training Aerobic HLY	Training Resistance HLY	Training Aerobic MTI	Training Resistance MTI
**Studies**	6	8	6	3
**Total females**	6	30	15	10
**Total males**	49	64	63	19
**Total undefined**	0	67	0	0
**%females**	10.9	31.9	19.2	34.5
**Age, mean±Sd**	50.7 ± 19.3	54.5 ± 23.2	48 ± 5.9	52.1 ± 13.8
**BMI, mean±Sd**	24.6 ± 1.7	25.7 ± 1.7	35.5 ± 6	34.8 ± 7.2
**GEOs**	GSE1786, GSE20319, GSE24215, GSE27543, GSE43760, GSE9103	EMEXP740, GSE106865, GSE16907, GSE28422, GSE47881, GSE8479, GSE97084, GSE117525	GSE1295, GSE40551, GSE43760, GSE48278, GSE58249, GSE72462	GSE48278, GSE58249, GSE117525
