# Supplementary material for: Transcriptomic profiling of skeletal muscle adaptations to exercise and inactivity
Source: Nat Commun. 2020 Jan 24;11:470. doi: 10.1038/s41467-019-13869-w (PMC6981202; doi:10.1038/s41467-019-13869-w)
Supplement: Supplementary file 4 — Description of Additional Supplementary Files [file 41467_2019_13869_MOESM4_ESM.pdf]

Title: Supplementary Data 1.
Description: Characteristics of all studies included in the MetaMEx database.
Transcriptomic studies in human skeletal muscle were collected from the GEO repository. The associated publications were combed for clinical characteristics of the study populations, exercise type, time and duration and transcriptomic platform and method used.
