# Supplementary material for: Transcriptomic profiling of skeletal muscle adaptations to exercise and inactivity
Source: Nat Commun. 2020 Jan 24;11:470. doi: 10.1038/s41467-019-13869-w (PMC6981202; doi:10.1038/s41467-019-13869-w)
Supplement: Supplementary file 5 — Reporting Summary [file 41467_2019_13869_MOESM5_ESM.pdf]

nature research

Corresponding author(s): Juleen R. Zierath

Last updated by author(s): Oct 31, 2019

Reporting Summary

Nature Research wishes to improve the reproducibility of the work that we publish. This form provides structure for consistency and transparency in reporting. For further information on Nature Research policies, see Authors & Referees and the Editorial Policy Checklist.

Statistics

For all statistical analyses, confirm that the following items are present in the figure legend, table legend, main text, or Methods section.

n/a	Confirmed	
☐	☒	The exact sample size (*n*) for each experimental group/condition, given as a discrete number and unit of measurement
☐	☒	A statement on whether measurements were taken from distinct samples or whether the same sample was measured repeatedly
☐	☒	The statistical test(s) used AND whether they are one- or two-sided<br>*Only common tests should be described solely by name; describe more complex techniques in the Methods section.*
☒	☐	A description of all covariates tested
☐	☒	A description of any assumptions or corrections, such as tests of normality and adjustment for multiple comparisons
☐	☒	A full description of the statistical parameters including central tendency (e.g. means) or other basic estimates (e.g. regression coefficient) AND variation (e.g. standard deviation) or associated estimates of uncertainty (e.g. confidence intervals)
☒	☐	For null hypothesis testing, the test statistic (e.g. *F*, *t*, *r*) with confidence intervals, effect sizes, degrees of freedom and *P* value noted<br>*Give P values as exact values whenever suitable.*
☒	☐	For Bayesian analysis, information on the choice of priors and Markov chain Monte Carlo settings
☒	☐	For hierarchical and complex designs, identification of the appropriate level for tests and full reporting of outcomes
☒	☐	Estimates of effect sizes (e.g. Cohen's *d*, Pearson's *r*), indicating how they were calculated

Our web collection on statistics for biologists contains articles on many of the points above.

Software and code

Policy information about availability of computer code

Data collection	Data collection was performed using R 3.5.2 (www.r-project.org). Micro array data was analyzed using the package oligo (https://bioconductor.org/packages/release/bioc/html/oligo.html). RNA sequencing data was analyzed using the package DESeq2 (https://bioconductor.org/packages/release/bioc/html/DESeq2.html)
Data analysis	Analyses were performed using either GraphPad Prism 8.1 software (GraphPad Software Inc.) or R 3.5.2 (www.r-project.org) with the package limma (https://bioconductor.org/packages/release/bioc/html/limma.html). Meta-analysis was calculated using the package metafor (http://www.metafor-project.org/doku.php)

For manuscripts utilizing custom algorithms or software that are central to the research but not yet described in published literature, software must be made available to editors/reviewers. We strongly encourage code deposition in a community repository (e.g. GitHub). See the Nature Research guidelines for submitting code & software for further information.

Data

Policy information about availability of data

All manuscripts must include a data availability statement. This statement should provide the following information, where applicable:

- Accession codes, unique identifiers, or web links for publicly available datasets
- A list of figures that have associated raw data
- A description of any restrictions on data availability

Original data used for the meta-analysis is publicly available on the GEO repository https://www.ncbi.nlm.nih.gov/geo. The curated database (MetaMEx) generated during the current study are available at www.metamex.eu

Field-specific reporting

Please select the one below that is the best fit for your research. If you are not sure, read the appropriate sections before making your selection.

☒ Life sciences ☐ Behavioural & social sciences ☐ Ecological, evolutionary & environmental sciences

For a reference copy of the document with all sections, see nature.com/documents/nr-reporting-summary-flat.pdf

Life sciences study design

All studies must disclose on these points even when the disclosure is negative.

Sample size	Cell culture experiments in primary human myotubes requires a minimum of three repeats. Experiments were we expected low amplitudes changes included more repeats to account for the variability due to the genetic variability accross cells from different donors.
Data exclusions	No data were excluded.
Replication	No replication tests were performed.
Randomization	In the meta-analysis, we did not control the study design of previously published studies. For the cell experiments, groups cannot be randomized.
Blinding	Blinding was not possible in cells experiments.

Reporting for specific materials, systems and methods

We require information from authors about some types of materials, experimental systems and methods used in many studies. Here, indicate whether each material, system or method listed is relevant to your study. If you are not sure if a list item applies to your research, read the appropriate section before selecting a response.

Materials & experimental systems

n/a	Involved in the study	
☐	☒	Antibodies
☒	☐	Eukaryotic cell lines
☒	☐	Palaeontology
☒	☐	Animals and other organisms
☐	☐	Human research participants
☒	☐	Clinical data

Methods

n/a	Involved in the study	
☒	☐	ChIP-seq
☒	☐	Flow cytometry
☒	☐	MRI-based neuroimaging

Antibodies

Antibodies used	NR4A3/NOR1 - NBP2-46246, Novus Biologicals (1:1000)<br>Total OXPHOS Human WB Antibody Cocktail - ab110411, Abcam (1:1000)<br>GAPDH - sc-25778, Santa Cruz Biotechnology (1:1000)
Validation	NR4A3 antibody was validated using siRNA-based silencing.

Human research participants

Policy information about studies involving human research participants

Population characteristics	Aerobic studies included 8 lean healthy men undergoing training aerobic exercise (Age 20 years; VO2max 45.1 ml x min−1 x kg −1) or acute aerobic exercise (Age 21 years; VO2max 46.7 ml x min−1 x kg−1). The resistance study included 8 healthy men (Age 27.4 years, VO2max 44.2 ml x min−1 x kg−1) undergoing 8 weeks of resistance training.
Recruitment	The samples were obtained at Victoria University, Melbourne, Australia.
Ethics oversight	Approval for all the experimental protocols and the study's procedures, which conformed to the standards set by the latest revision of the Declaration of Helsinki, was granted by the Victoria University Human Research Ethics Committee.

Note that full information on the approval of the study protocol must also be provided in the manuscript.
